# Understanding Mendelian errors in SNP arrays data using a Gochu Asturcelta pig pedigree: genomic alterations, family size and calling errors

**DOI:** 10.1038/s41598-022-24340-0

**Published:** 2022-11-16

**Authors:** Katherine D. Arias, Isabel Álvarez, Juan Pablo Gutiérrez, Iván Fernandez, Juan Menéndez, Nuria A. Menéndez-Arias, Félix Goyache

**Affiliations:** 1 Área de Genética y Reproducción Animal, SERIDA-Deva, Camino de Rioseco 1225, 33394 Gijón, Asturias Spain; 2 Departamento de Producción Animal, Universidad Complutense de Madrid, Avda. Puerta de Hierro s/n, 28040 Madrid, Spain; 3 ACGA, C/ Párroco José Fernández Teral nº 5 A, 33403 Avilés, Asturias Spain

**Keywords:** Genetics, Animal breeding, Genetic markers, Genotype

## Abstract

Up to 478 Gochu Asturcelta pig parents-offspring trios (61 different families) were genotyped using the Axiom_PigHDv1 Array to identify the causes of Mendelian errors (ME). Up to 545,364 SNPs were retained. Up to 40,540 SNPs gathering 292,297 allelic mismatches were identified and were overlapped with SINEs and LINEs (*Sscrofa* genome 11.1). Copy number variations (CNV) were called using PennCNV. ME were classified into eight different classes according to the trio member (“Trio” meaning no assignment) and the allele on which ME was identified: TrioA/B, FatherA/B, MotherA/B, OffspringA/B. Most ME occurred due to systematic causes: (a) those assigned to the Father, Mother or Offspring occurred by null or partial null alleles characterized by heterozygote deficiency, varied with family size, involved a low number of loci (6506), and gathered most mismatches (228,145); (b) TrioB errors varied with family size, covaried with SINEs, LINEs and CNV, and involved most ME loci (33,483) and mismatches (65,682); and (c) TrioA errors were non-systematic ME with no sampling bias involving 1.2% of mismatches only and a low number of loci (1939). The influence of TrioB errors on the overall genotyping quality may be low and, since CNV vary among populations, their removal should be considered in each particular dataset. ME assignable to the Father, Mother or Offspring may be consistent within technological platforms and may bias severely linkage or association studies. Most ME caused by null or partial null alleles can be removed using heterozygote deficiency without affecting the size of the datasets.

## Introduction

When using SNP arrays data, Mendelian Errors (ME) can be defined as the identification of variant calls inconsistent with the rules of Mendelian inheritance. Although ME can reflect true genomic variation arising from de novo mutations, in the case of correct pedigree information they more likely result from genotype calling errors. At least in humans, estimates of the de novo mutation rate are assumed to be ∼ 10^–8^ per locus^1,2^ while, in the literature, ME rates are substantially higher, often varying from 1.2 × 10^–3^ to 0.15 per locus^3,4^. Although not all genotyping errors result in Mendelian violation^5^, ME can be identified with certainty and factors contributing to their appearance, such as the presence of genomic alterations such as either short, SINEs, or long, LINEs, interspersed nuclear elements, studied^6^.

Genotyping errors due to technical issues can vary with the genotyping platform^7,8^. Furthermore, although joint academic-industrial efforts have developed algorithms successful in improving genotyping accuracy, their performance may be lower if systematic errors of the array reaction exist or the typed sites are located in genomic regions carrying copy number alterations^9,10^ usually referred to as Copy Number Variations (CNV). CNV are DNA segments ranging in size from 50 base pairs (bp) to several megabases (Mb) in which insertion, duplication or deletion events have occurred^11,12^. CNV modify the number of A and B alleles and, therefore, their intensities from the array causing misclassification. Actually, a successful genotyping approach should depend on the relative relationship between the A and B alleles from the array^10^. In Illumina SNP arrays, the alleles at each position are determined according to the intensity of a probe signal at a specific marker compared to the expected intensity, and the normalized B-allele frequency^13^. The genotyping software accompanying Affymetrix SNP arrays compares the allelic intensities detected for every SNP with those obtained for a number of probe quartets, each of which is composed of a 25-base-pair either matching the target sequence or not (on the 13th base) for alleles A and B separately using a Mahalanobis distance classifier under a Bayesian framework^14,15^.

ME assessment can be informative on the accuracy of variant calling pipelines^16,17^. Furthermore, the assessment of Mendelian Errors is considered useful to characterize the quality of genotype calling^3,6,18–20^. It may contribute to avoid undesirable effects on genetic analyses, such as reducing power in linkage and association studies and, particularly, causing confusion in studies aiming at the identification of rare variants or de novo mutations^6,21,22^.

The identification of ME needs the availability of reliable and dense pedigrees formed by a sufficient number of parent–offspring trios as well as the assumption that ME can result from diverse error mechanisms including the inheritance of the maternal allele^6,8^. The greater the number of closely related individuals is in a pedigree, the more power and the higher accuracy the pedigree allows for error detection and estimation^3^. Gochu Asturcelta is an extremely endangered pig breed kept in Asturias (Northern Spain) belonging to the Celtic pig strain of the Iberian Peninsula^23^. Recovery program allowed to obtain a complex pedigree^24,25^ useful to follow Mendelian inheritance across parent–offspring trios and within full-sib litters. This research uses genomic profiles of a sample of Gochu Asturcelta data to ascertain the extent to which different factors such as technological issues, family structure or genomic features including CNV and the presence of interspersed nuclear elements can affect calling quality causing ME. The importance of systematic and non-systematic errors in SNP arrays data and the need of implementing marker-based quality-control measures are discussed.

## Methods

### Samples and genotyping

**Figure 1:**
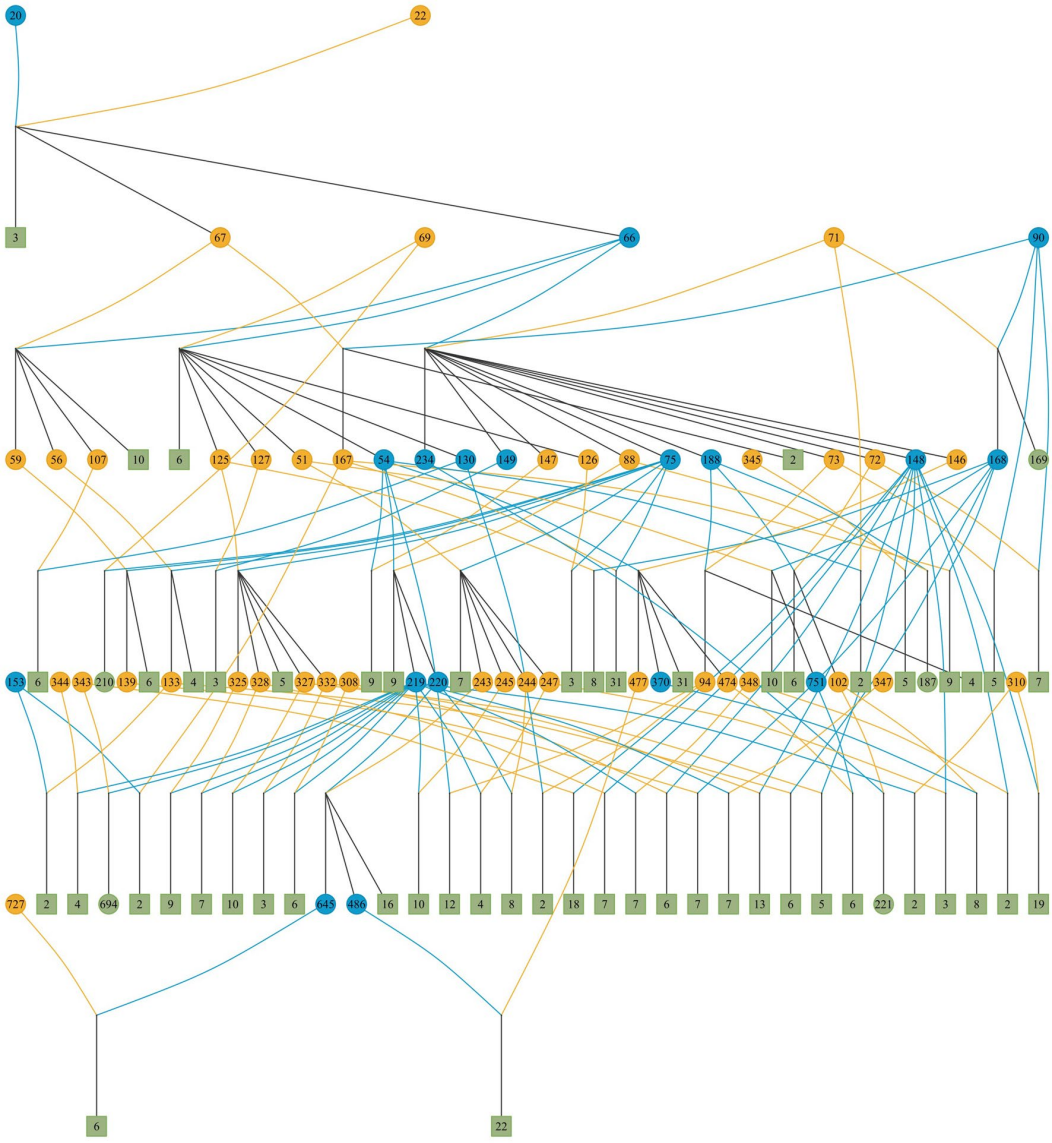
Gochu Asturcelta pig pedigree available. Numbers within circles are the identifications of the reproductive males (boars; in blue circles) and females (sows; in orange circles). Numbers within green squares inform on the offspring size of a parental couple excluded the offspring kept for reproduction. Green circles indicate that a given parental couple had one offspring only.

Data consisted of 492 Gochu Asturcelta individuals forming 478 parent–offspring trios that could be summarized into 61 different families (descendants of the same parental couple). Individuals were obtained from 96 registered litters (formed by two known parents and their offspring in a farrowing season). The available pedigree is illustrated in Fig. 1. Structure of data is fully described in Supplementary Table S1 and summarized in Table 1. Offspring genotypes derived from 15 genotyped boars and 28 genotyped sows.

**Table 1:** Structure of data.

Variable	Totals	With Mendelian errors (ME)
Number of individuals	492	478
Number of parent–offspring trios		478
Number of families		61
Number of boars		15
Number of sows		28
Number of litters		96
Mean offspring per family		7.8 [1; 34]
Mean offspring per litter		5.0 [1; 12]
Number of genotypes	545,364	40,304
Mean number of sites per autosome	30,298 [13,334; 62,927]	2239 [765; 5044]
Mendelian mismatches		292,297
Mean number of ME per individual		611.5 [331; 2016]
Number of CNV identified	3549	2262
Number of SNPs within CNV	56,737	13,359
Number of sites overlapping SINEs	26,676	2058
Number of sites overlapping LINEs	92,972	8889

Maximum and minimum values are in brackets.

Individuals were genotyped using the Axiom Porcine Genotyping Array (Axiom_PigHDv1) for pig genotyping^26^ containing assays for 658,692 SNPs with SNP positions based on *Sscrofa* genome build 11.1^27^. The software Axiom™ Analysis Suite v4.0.3 (Thermo Fisher Scientific, Waltham, MA) was used to create both standard genotypic .ped and .map files and intensity data useful for CNV calling. SNPs with ambiguous chromosome locations and SNPs located on either sexual chromosomes or mitochondrial DNA were excluded. Only animals with at least 95% of their SNPs called and individual SNPs with at least 99% call rate were considered. No thresholds for Minor Allele Frequency (MAF) or departures from Hardy–Weinberg (HW) proportions were applied to allow a correct identification of ME. Finally, a total of 545,364 SNPs with an average call rate of 99.79% were retained on the 18 *Sus scrofa* autosomes (SSC).

### Identification, quantification and annotation of Mendelian Errors

The program COLONY v.2.0.6.8^3,28^ was used to verify parentage in the pedigree.

SNPs and individuals showing ME were identified fitting the*—mendel* option of the program PLINK v.1.9. The software iterates through all trios and all variants checking for these errors^29^. Loci in which ME were identified were hereafter referred to as ‘ME set’. Considering the parental genotypes (either homozygous or heterozygous), the origin of the ME was assigned to the Father, the Mother, the Offspring, or the “Trio” when it was not possible to ascertain on which member of the trio occurred the genotype calling error. Moreover, considering the frequency of the allele on which the Mendelian violation was identified at a locus, ME was further classified as either A (occurring on the most frequent allele) or B (for the less frequent allele). Consequently, ME were classified into eight different classes: TrioA; TrioB; FatherA; FatherB; MotherA; MotherB; OffspringA; and OffspringB. These ME classes are expected to account for possible bias due to the sex of the parental allele inherited^8^ and the possible influence of changes in allele frequencies due to the genomic alterations^5,6^ in ME occurrence. Using the function—*hardy* of PLINK, heterozygote deficiency or excess (${F}_{IS}$) was computed for each locus in the ME set as ${F}_{IS}=\frac{{H}_{e}-{H}_{o}}{{H}_{e}}$, being ${H}_{e}$ and ${H}_{o}$ the expected and the observed heterozygosity, respectively.

Possible calling errors causing the appearance of either null alleles or false alleles on an SNP were also considered. SNPs included in the ME set were classified as Allele-Drop-Out (ADO; i.e. ‘missing’ alleles at a locus) when no heterozygote genotypes were identified in the whole typed population but homozygous genotypes existed for both the A and the B alleles. In turn, SNPs were classified as Allele-Drop-In (ADI; alleles that are additional to the parental genotypes) when: (a) all reproductive individuals were homozygous for the same allele but heterozygous genotypes were identified in the offspring (ADI_het_); and (b) all reproductive individuals were homozygous for the same allele but homozygous genotypes for the other allele were assessed in the offspring (ADI_hom_).

Following Pompanon et al.^4^ ME rates were quantified as: (a) Mean error rate per locus (${e}_{l}$) as ${e}_{l}=\frac{{m}_{l}}{nt}$; and (b) Mean error rate per allele (${e}_{a}$) computed as ${e}_{a}=\frac{{m}_{a}}{2nt}$, were ${m}_{l}$ is the number of single-locus genotypes including at least one allelic error, ${m}_{a}$, the number of allelic mismatches, and $nt$, the number of replicated single-locus genotypes.

The *intersectBed* function of the BedTools^30^ software was used to overlap the ME set with two categories of repeat elements: long interspersed nuclear elements (LINE) and short interspersed nuclear elements (SINE) using the *Sscrofa* genome build 11.1.

### CNV calling

The program PennCNV^31^ was used to perform CNV calling from the 18 autosomes of each individual in the dataset. PennCNV implements a hidden Markov model to detect CNV based on the log of the observed probe hybridization intensity divided by the expected probe hybridization intensity of SNPs (LRR) and the proportion of B alleles at an SNP (BAF). To adjust genomic waves, the—*gcmodel* option was used. Overlapping between the CNV and the SNPs having ME identified in each individual was assessed using the *intersectBed* function of the BedTools^30^ software.

### Basic statistical analyses and visualization of data

Gochu Asturcelta pedigree was visualized using the library visPedigree^32^ of R environment. Correspondence analyses aiming at the assessment of the relationships between the eight classes of ME, CNV alterations, and annotated repeat elements were performed using the library FactoMineR^33^ of R environment. Eigenvectors computed on each individual using correspondence analyses were used to construct bidimensional dispersion plots using the library ggplot2^34^ of R environment. Manhattan plots were constructed to illustrate data variation per chromosome and a count plot illustrating the relationships between the ME set loci and the eight classes of ME was created using the library ggplot2^34^ of R environment as well.

### Ethics declarations

SERIDA is adhered to the Ethical Committee in Research of the University of Oviedo (Spain) which ensures that all research with biological agents follows Good Laboratory Practices and European and Spanish regulations on biosecurity under the Regulation of February 13th, 2014 (BOPA no. 47on February 26th, 2014). In any case, blood and hair root samples used in this project were collected by veterinary practitioners working for the Gochu Asturcelta Breeders’ Association (ACGA), with the permission and in presence of the owners. For this reason, permission from the Ethical Committee in Research of the University of Oviedo was not required. In all instances, ACGA veterinarians followed standard procedures and relevant national guidelines to ensure appropriate animal care.

## Results

### Description of the ME set

**Figure 2:**
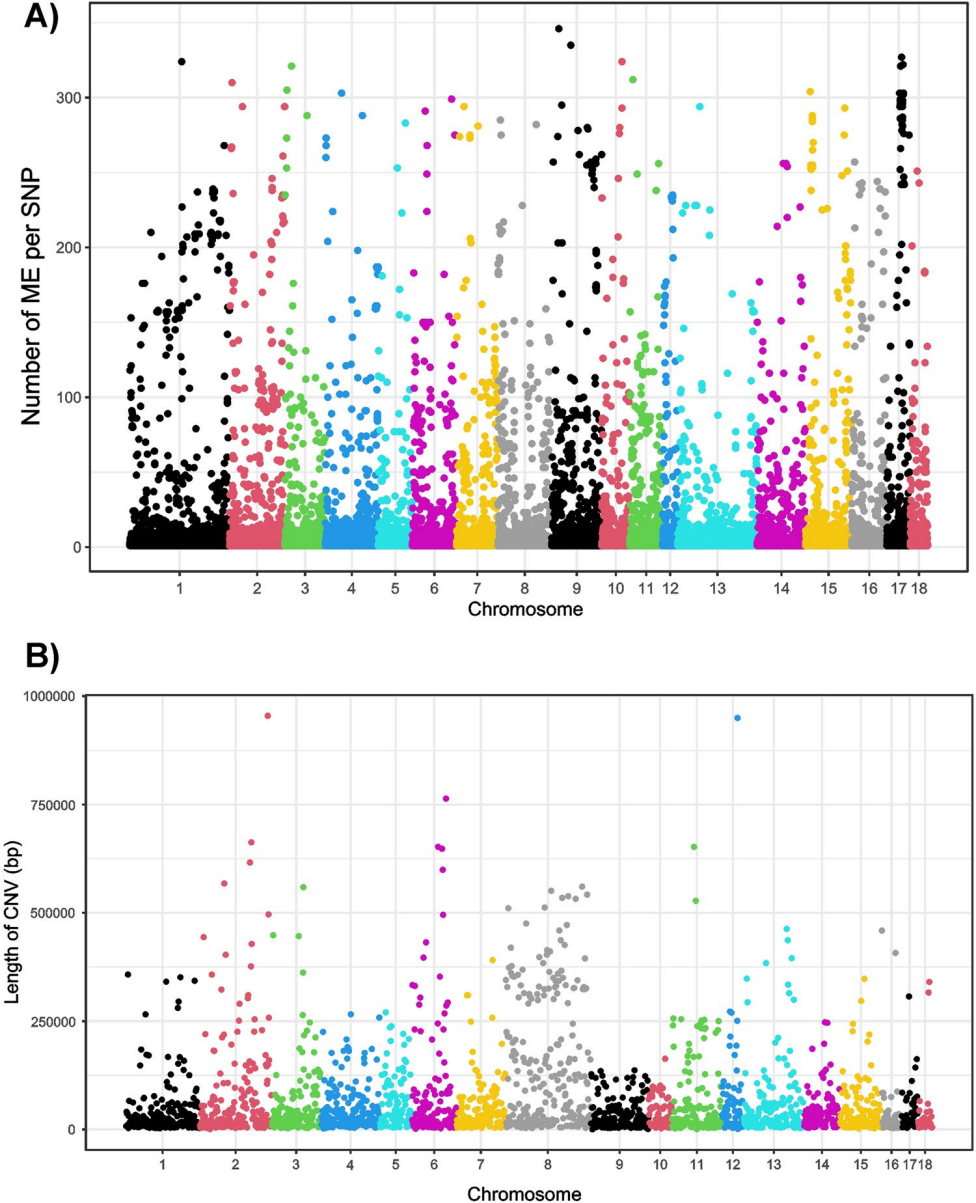
Manhattan plots illustrating: (**A**) the number of Mendelian Errors per SNP (on the Y-axis) and porcine chromosome (on the X-axis); and (**B**) the CNV identified on the individuals typed using the PennCNV calling platform (length of the CNV, in kb, is on the Y-axis).

Full data are given in Supplementary Table S1. A total of 40,540 SNPs (${e}_{l}$ = 0.074) gathering 292,297 allelic mismatches attributable to ME (${e}_{a}$ = 5.45 × 10^–4^) were identified. Up to 24,312 (60.3%) loci had inconsistent calls in a single trio (hereafter referred to as ‘unique’^6^), substantially contributing (0.0446) to the total mean error rate per locus. Up to 37,183 SNPs (66,303 mismatches) had ME in less than 10 individuals and 3,121 loci (225,994 mismatches) had ME in more than 10 individuals. Up to 293 loci gathered 200 ME or more and 17 of them had at least 300 mismatches (Supplementary Table S2; Fig. 2A). Most loci having ME were located on SSC1 (5044 SNPs -12.5%- and 35,690 mismatches -12.2%-) and SSC13 (4015 SNPs -10.0%- and 15,667 mismatches -5.4%-) whereas porcine autosomes 12, 17 and 18 gathered less than 3% of the loci included in the ME set (Supplementary Table S2).

ME, varying from 331 to 2016 per individual (611.5 per individual on average), were identified in the 478 parent–offspring trios available (Table 1; Supplementary Table S1). Family size varied considerably (from 1 to 34). The total number of mismatches per family had a clear covariation with family size (Supplementary Fig. S1) meaning that the higher the family size the higher the number of mismatches identified in a family.

### CNV and interspersed nuclear elements

PennCNV identified a total of 5,450 CNV across individuals summarized in 3549 CNV (Supplementary Table S3; Fig. 2B). Most of them (76.3%) were classified as duplications, whereas 16.5 and 6.5% of the CNV identified were classified as heterozygous and homozygous deletions, respectively. The CNV identified varied from 1 to 73 CNV per individual. Porcine autosomes 1, 2, 8, 9 and 13 carried 400 or more CNV (2445 in total). SSC16, 17 and 18 gathered less than 2% of the CNV identified each. Up to 2262 of the CNV identified (42%) spanned SNPs included in the ME set (Table 1). Altogether, CNV overlapped with a total of 13,359 (33%) of the loci of the ME set gathering 100,648 mismatches (Supplementary Table S2). SSC1, SSC13 and SSC4 gathered most of the ME loci located within CNV (23.4%; Supplementary Table S2).

A total of 8889 SNPs (22% of the total) included in the ME set, gathering 50,222 mismatches, were located within LINEs. These figures were lower for SINEs (2058 loci and 13,716 mismatches). Most ME loci located within interspersed nuclear elements were identified on porcine chromosomes 1, 9 and 2 (Supplementary Table S2).

### Characterization of the ME identified

Table 2 gives the frequency of the ME identified per error class. The origin of most mismatches identified could be assigned to either the Father (36.8% of the total) or the Mother (38.2%). Within parental classes, the number of mismatches identified on the A and the B alleles was well balanced, varying from 17.6% (FatherB class) to 19.3% (MotherB class) of the total. A total of 69,284 mismatches could not be assigned to any member of the parent–offspring trio. Failure in assignment mainly occurred for the less frequent allele with the TrioB class gathering 22.5% of the mismatches identified but it also had the lower mean mismatches per individual (3.43). Two-thirds (20,980) of the SNPs having ME classified as TrioB were unique. The proportion of mismatches assigned to Offspring was very low (1.2%) and mainly occurred on the more frequent allele (OffspringA class).

**Table 2:** ME frequency according to the eight ME classes defined.

ME class	Allele	All Mendelian errors					Frequency of Allele-Drop errors		
		Number of loci	Number of mismatches	Mean mismatches per individual	Mean families per ME loci	F_IS_	ADO	ADI_het_	ADI_hom_
Trio	A	1939	3602 (0.012)	11.9	4.12	− 0.011 (0.154)			
	B	33,483	65,682 (0.225)	3.43	1.97	− 0.044 (0.092)		1	
Father	A	3718	56,211 (0.192)	57.37	12.47	0.231 (0.368)	0.272		
	B	2580	51,490 (0.176)	75.91	15.54	0.311 (0.397)	0.219		
Mother	A	4304	55,103 (0.189)	50.38	11.16	0.191 (0.354)	0.232		
	B	3553	56,556 (0.193)	59.68	12.82	0.237 (0.374)	0.268		
Offspring	A	262	2224 (0.008)	103.31	22.61	0.500 (0.340)	0.004		
	B	325	1429 (0.005)	174.75	29.25	0.708 (0.312)	0.004		1
Total		50,164^b^	292,297			− 0.023 (0.157)	0.272		

The following information is given: number of loci having mismatches in each error class, number of mismatches (proportion of the total mismatches identified in brackets), mean number of mismatches per both individual and family, and mean *F*_*IS*_ (± standard deviation). Note that 4604 loci had mismatches assigned to two or more ME classes (see Fig. 4). Furthermore, the frequencies of Allele Drop-Out (ADO; 388 loci and 75,843 mismatches) and Allele Drop-In identified on heterozygotes (ADI_het_; 3447 loci and 5397 mismatches) or homozygotes (ADI_hom_; 17 loci and 37 mismatches) per error class are also given.

**Figure 3:**
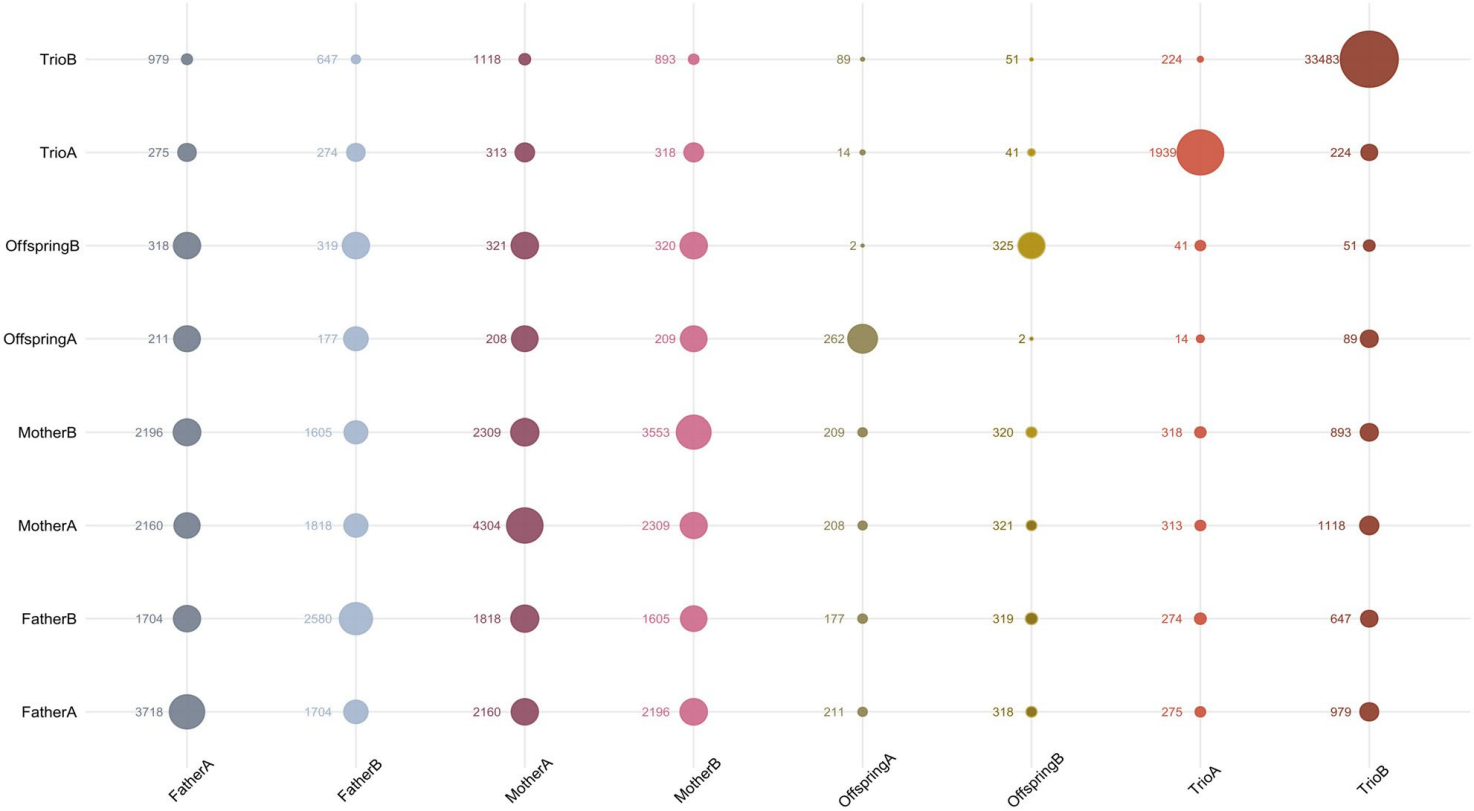
Geom-count plot illustrating the number of SNPs gathering Mendelian errors assigned to two-by-two of the eight ME classes defined according to the assignment of the origin of the error to a member of the parent–offspring trio and the frequency of the alleles at a locus.

In any case, 4604 loci (11.4% of the ME set) gathered 227,970 mismatches (78% of the total) that could be assigned to various ME classes. Figure 3 illustrates the frequencies of the ME loci assigned to two-by-two errors classes: on the bottom left corner, the loci having ME classified into both the Mother and the Father error classes (2837 loci and 215,105 mismatches) had a balanced representation in the other parental error classes, illustrating the high proportion of shared loci among such classes; furthermore, the two Trio error classes (on the upper right corner of Fig. 3) shared a low proportion of loci with other ME classes.

Although the TrioB error class gathered the higher number of both loci and mismatches in the ME set (Table 2), both the mean number of mismatches per individual (3.43) assigned to this class and the mean number of families in which such ME class could be identified (1.97) were the lowest in the ME set. On the contrary, errors classified into the OffspringA and OffspringB classes occurred in the larger number of families (22.61 and 29.25, on average, respectively). Furthermore, mismatches assigned to the Offspring error classes tended to accumulate in the same individuals (Table 2). Up to 87% of the loci having errors classified as OffspringA and all loci with ME assigned to the OffspringB class had mismatches assigned to the Father and the Mother classes (Fig. 3; Supplementary Table S2).

Most typed loci did not show either heterozygote excess or deficiency (Table 2). Only 2% of the loci belonging to the ME set had heterozygote excess (${F}_{IS}$ ≤ − 0.2; 796 SNPs). Up to 4% of them had heterozygote deficiency (${F}_{IS}$ ≥ 0.2; 1655 SNPs). ${F}_{IS}$ showed a marked variation between ME classes (Table 2). At the whole ME set level and within Trio error classes, loci did not show clear deviation on the expected number of heterozygotes with low and negative mean ${F}_{IS}$ values. However, the parental error classes tended to have heterozygote deficiency with positive ${F}_{IS}$ values ranging from 0.191 (MotherA) to 0.311 (FatherB). Up to 80% of the loci having ME assigned to the Father and Mother classes are in heterozygote deficiency (${F}_{IS}$ ranging from 0.1 to 1). This heterozygote deficiency scenario was even more marked for the Offspring error classes with mean ${F}_{IS}$ values varying from 0.500 (OffspringA) to 0.708 (OffspringB).

### Sources of variation of ME classes

**Figure 4:**
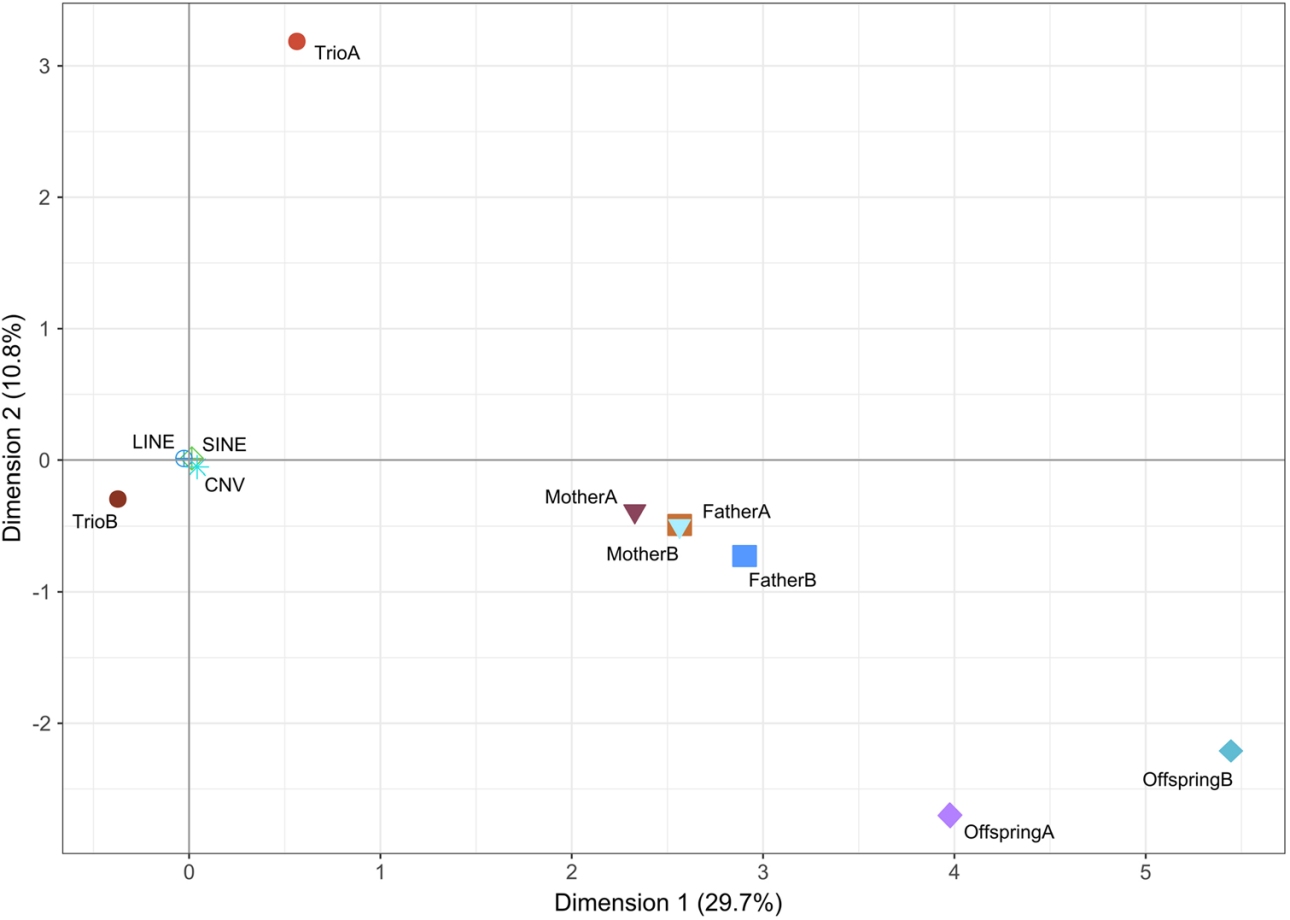
Dispersion plot constructed using the two canonical dimensions computed via corresponding analysis to illustrate the relationships between ME error classes, CNV, SINEs and LINEs. The Trio error classes are in circles, the Mother error classes are in triangles, the Father error classes are in squares, the Offspring error classes are in diamonds and CNV, SINEs and LINEs are in asterisks. Dimension 1 (on the X-axis) explained 29.7% of the variance and Dimension 2 (on the Y-axis) 10.8%.

Relationships between ME error classes and genomic features (CNV, SINEs, and LINEs) were summarized via correspondence analysis (Fig. 4). Dimension 1 (on the X-axis) separates the Offspring classes from the TrioB class while Dimension 2 (on the Y-axis) separates the TrioA class from the Offspring classes. Interestingly, the TrioB class covariates with CNV, SINEs, and LINEs. In turn, the four parental error classes covariated on the X-axis.

**Figure 5:**
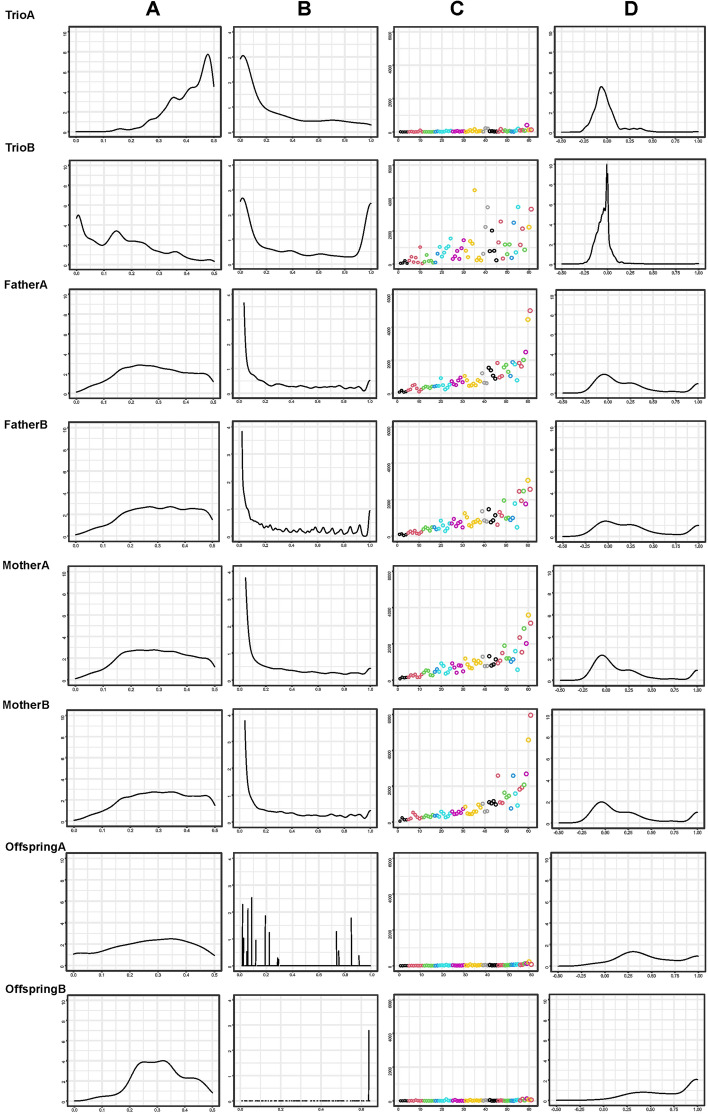
Plots characterizing the SNPs included in the ME set according to the eight classes of ME defined (TrioA; TrioB; MotherA; MotherB; FatherA; FatherB; OffspringA; and OffspringB). Density plots of the minor allele frequency (MAF) are shown in column (**A**); density plots of the probability (*p*) of the deviation of the Hardy–Weinberg proportions (HW) are shown in column (**B**); dispersion plots constructed according to the family size (on the X-axis) and the number of ME identified per family (on the Y-axis) are illustrated in column (**C**); finally, density plots of the ${F}_{IS}$ variation are shown in column (**D**).

Figure 5 shows density plots illustrating the variation of the minor allele frequency (column A) and the statistical probability (*p*) of deviation from the Hardy–Weinberg proportions (column B) for the eight classes of ME defined. Except for the TrioB class in which the minor allele tended to be in low frequency, MAF tended to be moderate. Loci having ME assigned to either the Father or the Mother tended to be in HW disequilibrium. However, this could not be assessed for either the Trio or the Offspring error classes, which mainly included loci in HW equilibrium. This pattern was the same for the variation of ${F}_{IS}$ within error classes: Parental and Offspring error classes had a marked heterozygote deficiency whereas the Trio error classes were formed by loci with ${F}_{IS}$ values near 0. Furthermore, Fig. 5 shows (in column C) dispersion plots constructed according to the family size (on the X-axis) and the number of ME identified per family (on the Y-axis). Following the general pattern (Supplementary Fig. S1), the identification of ME is biased due to family size in most error classes. However, the Offspring error classes and, particularly, the TrioA class departed from this expectation.

### Allele Drop-Out and Allele-Drop-In

A total of 388 loci (1% of those included in the ME set) which gathered 75,843 mismatches (26% of the total) were classified as ADO (Supplementary Table S2). In general, the ADO loci had MAF values varying from moderate to high (average MAF = 0.216), were in high HW disequilibrium with a strong deficiency of heterozygotes (${F}_{IS}$ = 1.0) and were mainly classified into the four parental (FatherA, FatherB, MotherA and MotherB) error classes (Table 2). ADO loci had no Trio mismatches. Up to 3447 loci (gathering 5397 mismatches) were classified as ADI_het_. Seventy-seven percent of them (2648) were unique. All ADI_het_ loci were in HW equilibrium with no deviation of excess or deficiency of heterozygotes (${F}_{IS}$ = − 0.002), had very low MAF (3415 loci with MAF below 0.01), and belonged to the TrioB error class. Up to 48% of the ADI_het_ loci did not map into CNV, SINEs or LINEs. Only 17 loci (gathering 37 mismatches) were classified as ADI_hom_. Although MAF was very low as well (ranging from 0.008 to 0.002), their behavior departed from that of the ADI_het_ loci: all ADI_hom_ loci significantly departed from the HW proportions with high heterozygote deficiency (${F}_{IS}$ = 1.0) and were classified into the OffspringB error class.

## Discussion

The occurrence of ME may depend on different genomic and non-genomic causes, such as differences between genotyping platforms^8,10,14^, the presence of genomic alterations such as CNV, SINEs or LINEs^6,10^, the allelic frequency and the size of the offspring typed^18,21^. Furthermore, the assessment of ME can be carried out using different statistics^3^, linkage disequilibrium^22^, or direct observation^6^. Here, we split the observed ME into different error classes to give new insights into the causes of the presentation of ME in SNP arrays data.

Identification of ME in SNP arrays data partially depends on sample size (here family size; Supplementary Fig. S1). Therefore, since our data is not completely independent because they were obtained within a relatively little number of families varying in size, the variation in ME occurrence summarized in Fig. 4 may have some bias. However, three different ME classes (OffspringA, OffspringB and TrioA) were not dependent on family size (Fig. 5) and, therefore, the correspondence analysis (Fig. 4) added to the characterization of the causes of ME. However, the Offspring error classes do not show a clear pattern of variation due to sampling. According to literature, sample size is expected to affect the possibility of identifying ME in the offspring^18,35^: ME identification would be more frequent when the error occurs in a parent than in the offspring unless the number of typed offspring is large. However, despite families forming our data set were mainly bigger than those usually available in humans for similar studies^36,37^, our results suggest that the identification of ME in the Offspring is rare and probably dependent on the systematic causes underlying the identification of ME in the parents.

Furthermore, literature suggests that identification of ME should be more problematic when the frequency of the minor allele is low^18,35^. Our results depart from this expectation except for the case in which the ME could not be assigned to a member of the parent–offspring trio (TrioB). Loci classified into the TrioB error class had a particular behavior within the ME set: although they involve a large number of loci, both the number of mismatches per loci and the number of individuals having such errors at a given locus were very low. An extreme case of TrioB errors are the ADI_het_ loci in which one allele is fixed in the individual with offspring in data and the ME occur in heterozygous offspring with a “new” allele added to the parental genotypes. Furthermore, TrioB errors do not alter HW equilibrium and ${F}_{IS}$ at the locus level. On the contrary to the TrioA errors, the probability of identifying TrioB ME increases with sample size. The latter suggests that ME assigned to the TrioB class occur due to systematic causes (Fig. 4). SINEs and LINEs, which are enriched within poly-A/T sequences, and CNV can cause differences in calling quality between the members of the parent–offspring trio^6,10^, therefore hindering the correct assessment of the allelic frequencies of an SNP^38^. Our results suggest that such genomic features can underlie the occurrence of TrioB errors^6,10^. Furthermore, the influence of this type of genotyping errors may be variable among datasets^38^. Although SINEs and LINEs may have a general influence on genotyping quality within species, CNV may not. Moreover, CNV are assumed to mirror particular population histories^39^ and their importance and influence on the occurrence of ME should probably be assessed for each particular genotyping project unless sufficient evidence exists for a particular CNV in a species.

The behavior of the ME assigned to either the Father or the Mother paths (Figs. 3 and 4) suggests that parental error classes share the causes underlying these ME. Such error classes have balanced allele frequencies but tend to have significant deviations of the Hardy–Weinberg proportions and heterozygote deficiency (Fig. 5). In our data, a significant part of the mismatches assigned to parental error classes occur in loci having allele amplification difficulties (null alleles; ADO). They gathered the larger number of mismatches (Fig. 2A). Although the number of ADO loci is very low due to the conservative criterion used for its definition, it may not be discarded that many other loci having ME assigned to either the Father or the Mother could be ‘partial nulls’, widely characterized in microsatellite data sets^40,41^, i.e. alleles with no fully codominant signal that do not always generate missing data. The ascertainment between null and partial null alleles may be difficult when sample size is small^42^. Furthermore, our results suggest that ME assigned to Offspring can be extreme cases of calling problems also affecting to ME assigned to the parental (Father and Mother) classes: the number of ME assigned to the offspring is very low and most loci with OffspringA and OffspringB errors have mismatches on the parental error classes (Fig. 3). The main difference between the Offspring and the parental ME classes consists on that the Offspring error classes have the higher mean heterozygote deficiency (Table 2). ME assigned to either parental or offspring error classes may be consistent among populations within a given technological platform and, if kept in datasets used for either linkage or association studies, results may be severely biased.

Finally, ME assignable to the TrioA error class do not follow a distinguishable pattern of variation. TrioA errors occurred in a very low number of loci gathering the lowest number of mismatches per locus (1.86) and the lower number of total mismatches (1.2%). They are in loci with the minor allele having moderate to high frequency, mainly in HW equilibrium, most of them with no heterozygote excess or deficiency, and no clear sampling bias (Fig. 5). The ascertainment of the causes of the TrioA errors is not straightforward and, in general, could be considered non-systematic ME.

Since ME do not occur by chance, researchers routinely incorporate marker-based quality control measures to limit for spurious findings. These measures frequently consist in removing SNPs with MAF lower than 0.05 and those that did not adjust to the HW expectation with a threshold (*p* value) of ≤ 0.001 (e.g., Manunza et al.^43^). However, applying such conventional quality filters may not always be advisable because of their major impact on the final number of loci gathered for analyses^6^. The relevant subject is how these standard control measures affect the information provided by SNPs that do not gather ME.

From our data, fitting MAF ≤ 0.05 as a threshold cannot be recommended (Supplementary Fig. S2): the removal of the 6.4% of the ME mismatches would imply the removal of roughly a third (31.7%) of the total SNP set available (173,032 SNPs). The use of HW test for quality control in SNP arrays data is not usually recommended because of its low robustness and low statistical power. However, deviation of HW equilibrium applies to particular errors characterized by excess of homozygosity^3^. In our data, the use of two different HW thresholds (*p* values ≤ 0.001 or ≤ 0.0001) would imply the removal of a higher number of mismatches (70 and 66.7%, respectively) than with using MAF and a lower number of total SNPs (9 and 4.5%, respectively). In any case, the combined use of MAF (≤ 0.05) and HW thresholds (*p* < 0.001) would be inadvisable: although this strategy allows to remove the highest number of mismatches (222,422; 76%), it is done to the cost of removing the 40.8% of the SNPs (222,345) of the total dataset.

On the contrary to the TrioB errors, ME assigned to a member of the trio with certainty gather a high number of systematic mismatches Therefore, null and partial null alleles can lead to the identification of spurious associations or linkage disequilibrium segments. The removal of such errors (or at least those we defined as ADO) appears necessary^44^.

**Figure 6:**
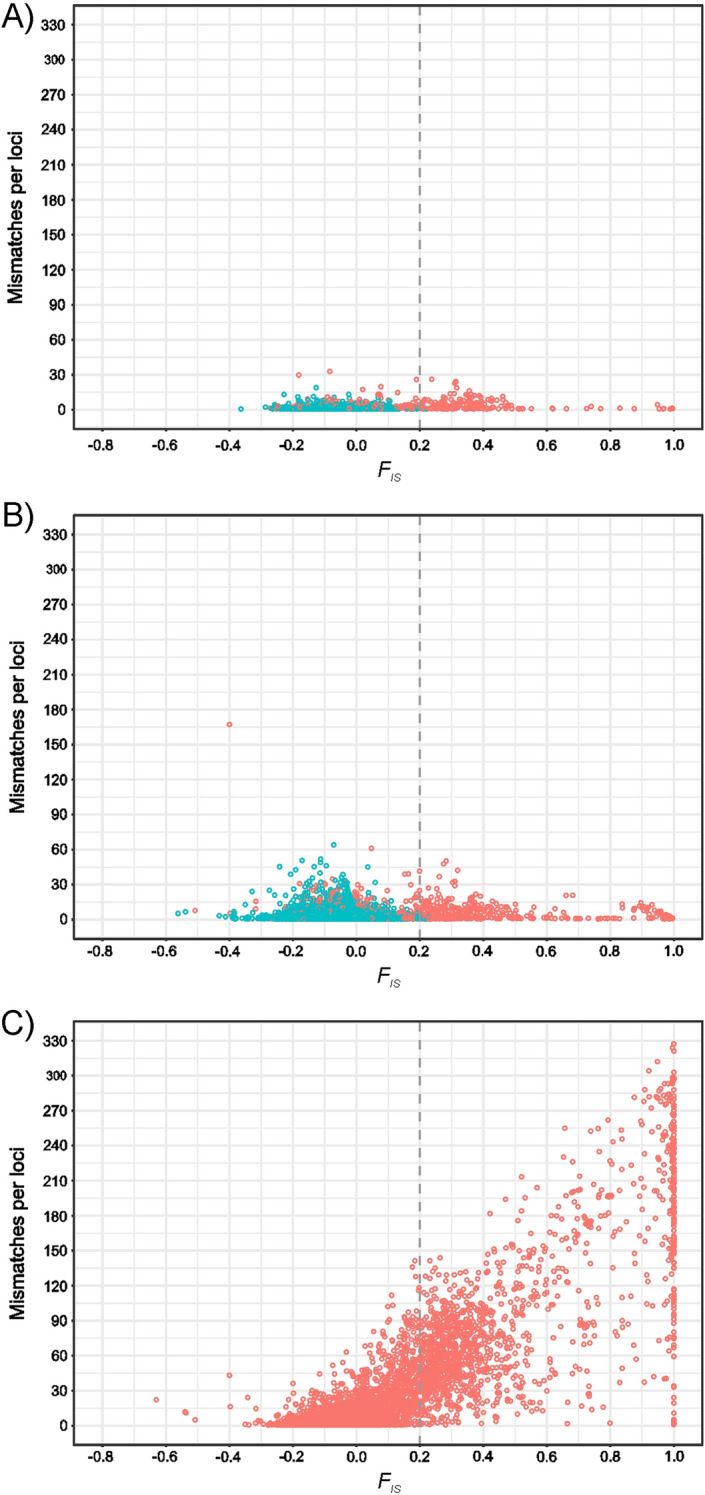
Number of allelic mismatches (on the Y-axis) per loci illustrated according to ${F}_{IS}$ value (on the X-axis) and classes of ME. Plots (**A**), (**B**) and (**C**) show the TrioA mismatches, the TrioB mismatches, and the sum of the mismatches assigned to the Father, Mother or Offspring error classes, respectively. Loci that have either TrioA or TrioB errors only are in blue whereas loci having errors assigned to a member of the trio (Father, Mother or Offspring) as well are in red. Vertical lines represent ${F}_{IS}$ threshold of 0.2.

Our results suggest that removal of loci having ME caused by null or partially null alleles (i.e., ADO and ADO-like alleles) should be approached before using SNP arrays data for further analyses. Since null genotypes could leave ‘footprints’ in SNP genotype data, including deviations from Mendelian inheritance and intense deviation from Hardy–Weinberg proportions^45^, they can affect the quality of the SNP arrays data to a large extent. Approaching this via HW tests may cause the removal of an undesirable number of truly informative loci. The use of filters based on heterozygote deficiency can be considered an alternative. However, since differences in heterozygote deficiency can be caused by either extensive natural selection or inbreeding as well^42,44^, this should be considered with caution due to Allele Drop errors only affect only a subset of loci^41^. Mismatches assessed in our dataset are clearly biased to positive ${F}_{IS}$ values (Fig. 6). Therefore, applying thresholds for ${F}_{IS}$ ≥ 0.2 allows to remove a significant proportion of mismatches (64%; 187,213) with the removal of a very small proportion of SNPs in the whole dataset (0.4%; 2041), most of them (1656) being loci included in the ME set (Supplementary Fig. S2).

In summary, our results contribute to the understanding of the causes and importance of Mendelian Errors in SNP arrays data confirming that all ME cannot be considered the same and that most ME probably occur due to systematic causes^6,46^. Classification of ME into eight different error classes and the characterization of three Allele Drop sources of errors allow us to suggest that there are two main causes of most ME: (a) the presence at the locus location of genomic alterations such as CNV, SINEs and LINEs; and (b) the presence of null and partial null alleles. The presence of null alleles, although involving a limited number of loci affects a significant number of genotypes and can be challenging for association and linkage studies and the identification of rare variants in a genome. ME due to genomic alterations, however, can be particular for each population under study and may not have a strong influence on the results of either association or diversity analyses. Using ${F}_{IS}$ as a quality criterion for SNP arrays data may be enough to remove most mismatches due to the presence of null and partial null alleles.

## Supplementary Information

Supplementary Information.

## Data Availability

All data obtained are provided as Supplementary Tables and Figures (.xlsx file).

## References

[1] Veltman JA, De Brunner HG (2012). novo mutations in human genetic disease. Nat. Rev. Genet..

[2] Wong WSW (2016). New observations on maternal age effect on germline de novo mutations. Nat. Commun..

[3] Wang J (2018). Estimating genotyping errors from genotype and reconstructed pedigree data. Methods Ecol. Evol..

[4] Pompanon F, Bonin A, Bellemain E, Taberlet P (2005). Genotyping errors: Causes, consequences and solutions. Nat. Rev. Genet..

[5] Li H (2014). Toward better understanding of artifacts in variant calling from high-coverage samples. Bioinform. Oxf. Engl..

[6] Kothiyal P, Wong WSW, Bodian DL, Niederhuber JE (2019). Mendelian inconsistent signatures from 1314 ancestrally diverse family trios distinguish biological variation from sequencing error. J. Comput. Biol..

[7] von Thaden A (2017). Assessing SNP genotyping of noninvasively collected wildlife samples using microfluidic arrays. Sci. Rep..

[8] Goldmann JM (2018). Germline de novo mutation clusters arise during oocyte aging in genomic regions with high double-strand-break incidence. Nat. Genet..

[9] Miyagawa T (2008). Appropriate data cleaning methods for genome-wide association study. J. Hum. Genet..

[10] Yang S, Cui X, Fang Z (2014). BCRgt: A Bayesian cluster regression-based genotyping algorithm for the samples with copy number alterations. BMC Bioinform..

[11] Feuk L, Carson AR, Scherer SW (2006). Structural variation in the human genome. Nat. Rev. Genet..

[12] Scherer SW (2007). Challenges and standards in integrating surveys of structural variation. Nat. Genet..

[13] Ritchie ME, Liu R, Carvalho BS, Irizarry RA, The Australia and New Zealand Multiple Sclerosis Genetics Consortium (ANZgene) (2011). Comparing genotyping algorithms for Illumina’s Infinium whole-genome SNP BeadChips. BMC Bioinform..

[14] Rabbee N, Speed TP (2006). A genotype calling algorithm for affymetrix SNP arrays. Bioinformatics.

[15] Affymetrix. BRLMM-P: A genotype calling method for the SNP 5.0. 16 (2007).

[16] Utsunomiya, Y. T., Alonso, R. V., Vinsintin, J. A. & Garcia, J. F. mendelFix: A Perl script for checking Mendelian errors in high density SNP data of trio designs (2013).

[17] Kómár P, Kural D (2018). geck: Trio-based comparative benchmarking of variant calls. Bioinformatics.

[18] Douglas JA, Skol AD, Boehnke M (2002). Probability of detection of genotyping errors and mutations as inheritance inconsistencies in nuclear-family data. Am. J. Hum. Genet..

[19] Geller F, Ziegler A (2002). Detection rates for genotyping errors in SNPs using the trio design. Hum. Hered..

[20] Khan SA (2017). Rules for resolving Mendelian inconsistencies in nuclear pedigrees typed for two-allele markers. PLOS ONE.

[21] Li B (2012). A likelihood-based framework for variant calling and de novo mutation detection in families. PLoS Genet..

[22] Wang RJ, Radivojac P, Hahn MW (2021). Distinct error rates for reference and nonreference genotypes estimated by pedigree analysis. Genetics.

[23] Menéndez J (2016). Genetic characterisation of the endangered Gochu Asturcelta pig breed using microsatellite and mitochondrial markers: Insights for the composition of the Iberian native pig stock. Livest. Sci..

[24] Menendez J, Alvarez I, Fernandez I, Goyache F (2016). Genealogical analysis of the Gochu Asturcelta pig breed: Insights for conservation. Czech J. Anim. Sci..

[25] Menéndez J, Álvarez I, Fernandez I, Menéndez-Arias NA, Goyache F (2016). Assessing performance of single-sample molecular genetic methods to estimate effective population size: Empirical evidence from the endangered *Gochu Asturcelta* pig breed. Ecol. Evol..

[26] Groenen, M. Development of a high-density Axiom® porcine genotyping array to meet research and commercial needs. In *Plant & Animal Genome XXIII Conference, San Diego, USA* (2015).

[27] Groenen MAM (2012). Analyses of pig genomes provide insight into porcine demography and evolution. Nature.

[28] Wang J (2019). Pedigree reconstruction from poor quality genotype data. Heredity.

[29] Chang CC (2015). Second-generation PLINK: Rising to the challenge of larger and richer datasets. GigaScience.

[30] Quinlan AR, Hall IM (2010). BEDTools: A flexible suite of utilities for comparing genomic features. Bioinformatics.

[31] Wang K (2007). PennCNV: An integrated hidden Markov model designed for high-resolution copy number variation detection in whole-genome SNP genotyping data. Genome Res..

[32] Luan, S. visPedigree. *visPedigree: A package for tidying and drawing animal pedigree*. https://github.com/luansheng/visPedigree (2018).

[33] Lê S, Josse J, Husson F (2008). FactoMineR: An R package for multivariate analysis. J. Stat. Softw..

[34] Wickham H (2016). ggplot2: Elegant Graphics for Data Analysis.

[35] Gordon D, Heath S, Ott J (1999). True pedigree errors more frequent than apparent errors for single nucleotide polymorphisms. Hum. Hered..

[36] Ewen KR (2000). Identification and analysis of error types in high-throughput genotyping. Am. J. Hum. Genet..

[37] Saunders IW, Brohede J, Hannan GN (2007). Estimating genotyping error rates from Mendelian errors in SNP array genotypes and their impact on inference. Genomics.

[38] Lee S, Kasif S, Weng Z, Cantor CR (2008). Quantitative analysis of single nucleotide polymorphisms within copy number variation. PLoS ONE.

[39] Fontanesi L (2011). A first comparative map of copy number variations in the sheep genome. Genomics.

[40] Dakin EE, Avise JC (2004). Microsatellite null alleles in parentage analysis. Heredity.

[41] Dewoody J, Nason JD, Hipkins VD (2006). Mitigating scoring errors in microsatellite data from wild populations. Mol. Ecol. Notes.

[42] Abramovs N, Brass A, Tassabehji M (2020). Hardy-Weinberg equilibrium in the large scale genomic sequencing era. Front. Genet..

[43] Manunza A (2016). A genome-wide perspective about the diversity and demographic history of seven Spanish goat breeds. Genet. Sel. Evol..

[44] Waples RS (2015). Testing for Hardy-Weinberg proportions: Have We lost the plot?. J. Hered..

[45] McCarroll SA (2006). Common deletion polymorphisms in the human genome. Nat. Genet..

[46] Neale BM, Purcell S (2008). The positives, protocols, and perils of genome-wide association. Am. J. Med. Genet. Part B Neuropsychiatr. Genet..

